# Inhibition of IGF1R in Early MMTV-Wnt1 Mammary Tumors: A Transcriptomic Analysis

**DOI:** 10.3390/cancers18111749

**Published:** 2026-05-27

**Authors:** Joseph J. Bulatowicz, Alexander Lemenze, Elvan Dogan, Christopher A. Galifi, Krystopher Maingrette, Quan Shang, Teresa L. Wood

**Affiliations:** 1 Department of Pharmacology, Physiology, & Neuroscience, Center for Cell Signaling, Cancer Institute of New Jersey, New Jersey Medical School, Rutgers Health, Newark, NJ 07103, USA; jjb353@njms.rutgers.edu (J.J.B.);; 2 Molecular and Genomics Informatics Core (MaGIC), New Jersey Medical School, Rutgers Health, Newark, NJ 07103, USA

**Keywords:** IGF1R, Wnt1, metastasis, differentiation, keratin, signaling, scRNA-seq

## Abstract

**Background**: The insulin-like growth factor 1 receptor (IGF1R) is a receptor tyrosine kinase whose both overexpression and underexpression have been implicated in the initiation and progression of breast tumorigenesis. The mechanism through which underexpression of the receptor contributes to a more aggressive phenotype is currently less understood. **Methods**: Through the expression of a dominant-negative IGF1R, we studied the phenotypic effects of receptor inhibition on early MMTV-*Wnt1* mouse mammary tumors. Utilizing histopathological techniques and single-cell RNA-sequencing, we explored cellular heterogeneity and transcriptional alterations that occur as a result of IGF1R inhibition. **Results**: Examination of primary tumors failed to reveal obvious differences in tissue architecture or expression of differentiation markers with IGF1R inhibition. Both cohorts of tumors produced metastatic lesions in the lung. Single-cell RNA-sequencing identified previously unknown epithelial subpopulations that were present in both tumor types. In tumors with inhibited IGF1R, a previously undescribed epithelial population marked by expression of both *Krt14* and *Krt6a* was identified, transcriptionally distinct from its MMTV-*Wnt1* counterpart, and present in the smallest lung metastases. In human breast cancer patients, expression levels of *KRT14* and *KRT6A* negatively correlated with expression of *IGF1R*. **Conclusions**: Inhibition of the IGF1R in a mouse model of basal-like breast cancer produces transcriptionally distinct *Krt6a^+^*/*Krt14^+^* epithelial cells, which are present in the smallest metastatic lesions identified in the lung. Expression of genes associated with this population may potentially be effective biomarkers of metastatic capacity in basal-like breast tumors with low levels of IGF1R expression.

## Simple Summary

Basal-like breast cancers are aggressive and difficult to treat clinically. Tumors of this subtype have decreased expression of the insulin-like growth factor 1 receptor (IGF1R). The aim of this study was to determine phenotypic changes that occur in basal-like tumors when IGF1R signaling is reduced. Transcriptomic analysis identified previously unknown cell subpopulations and alterations in gene expression that resulted from inhibition of the IGF1R in a mouse model of basal-like breast cancer. These outcomes can be used as biomarkers to enable new therapeutic approaches in patients with basal-like breast cancer.

## 1. Introduction

According to the World Health Organization, cancer is a leading cause of premature death, second only to cardiovascular disease [1]. In the most recent analysis of global statistics published in 2024 that analyzed data from 2022, it was revealed that female breast cancer was the most common cancer type in women (~2.3 million, 23.8% of all new cases) and was the leading cause of cancer-related mortality (0.66 million, 15.4% of all new deaths) [2]. Due to its heterogeneous nature, breast cancer can be classified into molecularly distinct subtypes (normal-like, luminal-A, luminal-B, Erb-B2 receptor tyrosine kinase 2^+^ (HER2^+^), and basal-like/triple negative), distinguished by their respective transcriptomic profiles [3,4,5,6]. Basal-like breast cancer (BLBC) and triple-negative breast cancer (TNBC) are often used interchangeably in the literature, yet mounting evidence suggests that although there are similarities between them, there exists a significant percentage of each that is phenotypically distinct from the other (for review, see [7]). Further molecular characterization of human patient samples immunohistochemically classified as TNBC has demonstrated that “triple negativity” is a potential feature of all currently known molecular subtypes; however, ~70% of samples analyzed presented as transcriptionally basal-like [8].

Basal-like/TNBCs account for 15–20% of all breast cancer diagnoses and are the most aggressive subtype with the lowest 5-year survival rate of ~77% [9,10]. The vast majority of breast cancer-related deaths (~60–90%) are attributed to metastasis, or the distant dissemination of cancer cells from the primary tumor to secondary organs [11,12]. Recently, the basal marker keratin 14 (KRT14) was identified as a key regulator of collective invasion and metastasis in the mouse mammary tumor virus (MMTV)-polyoma virus middle-T antigen (*PyMT)* breast cancer mouse model [13,14]. Although the transcriptomic profile of MMTV-*PyMT* tumors most closely resembles the human luminal-B subtype, subsequent lineage tracing experiments demonstrated that basal-like cells within these tumors, determined by positive expression of keratin 5 (KRT5) and KRT14, are derived from luminal cells that have gained basal characteristics [15,16]. These observations highlight the importance of the basal phenotype in metastatic progression. Furthermore, since most basal-like tumors lack expression of estrogen receptor, progesterone receptor, and HER2, common neoadjuvant therapies targeting these receptors are ineffective, necessitating the development of novel treatment options.

Canonical Wnt/beta (β)-catenin signaling is a commonly dysregulated pathway in a number of different cancers, including colorectal, lung, and breast [17,18,19]. Immunohistochemical and complementary deoxyribonucleic acid (cDNA) microarray analyses of human breast tumor tissue revealed a statistically significant association between β-catenin nuclear localization and estrogen receptor (ER) negativity, loss of E-cadherin, and a basal-like phenotype, characteristics shared with MMTV-*PyMT* tumors [20,21]. Importantly, the MMTV-Wnt family member 1 (*Wnt1*) breast tumor model, where overexpression of *Wnt1* in the mammary epithelium is sufficient to induce tumorigenesis, also expresses a transcriptomic profile consistent with that of the basal-like molecular subtype [15,22,23,24]. Additionally, it has been shown that the MMTV-*Wnt1* mouse model produces two phenotypically distinct tumors, distinguished by latency [25]. Early Wnt (eWnt1) tumors arise before fifteen weeks of age, are Wnt signaling-driven (unlike late tumors), and contain a majority population of cluster of differentiation 49f^+^ (CD49F^+^)/epithelial cell adhesion molecule^−^ (EPCAM^−^) cells, which are analogous to stem-like cells within the normal mammary gland [25]. Recently, the stem-like profile of eWnt1 tumors was independently validated by Spina et al., who demonstrated that early tumors contain large populations of bipotent, adhesion G-protein coupled receptor A3 (*Adgra3)*-expressing cells that exist within the cap cell layer of terminal end buds present in embryonic and pubertal mammary glands [26].

The insulin-like-growth factor 1 receptor (IGF1R) pathway possesses both oncogenic and tumor-suppressive functions in the context of breast tumorigenesis (for review, see [27]). Briefly, overexpression or constitutive activation of the receptor is sufficient to induce highly proliferative mammary tumors, whereas inhibition of IGF1R also results in a more aggressive tumor phenotype [28,29,30]. By inhibiting signaling through expression of a human dominant-negative IGF1R in the MMTV-*Wnt1*/*dnIGF1R* transgenic mouse, our lab previously demonstrated that blocking IGF1R in basal-like breast cancer decreases tumor latency, enhances the tumor basal phenotype, and increases metastasis to the lung [30]. Moreover, these tumors contain alterations to their immune infiltration, microenvironment, and adhesive capabilities, contributing to their metastatic phenotype [31,32]. Unfortunately, the conclusions of these studies investigating the effect of IGF1R inhibition on MMTV-*Wnt1* tumors were limited by the absence of accountability for the dual latency phenotypes that have since been identified in this model [25].

Here, we investigated the hypothesis that MMTV-*Wnt1*/*dnIGF1R* mice produce early (eDN-Wnt1) tumors containing transcriptionally altered cells within the luminal compartment that have gained basal characteristics. We performed single-cell ribonucleic acid-sequencing (scRNAseq), which revealed a number of epithelial populations not previously described in eWnt1 tumors, as well as transcriptional alterations to these cells in the luminal compartment as a result of IGF1R inhibition. Immunohistochemical analysis of the smallest identified metastatic lesions identified a population of cells marked by co-expression of the bipotential progenitor marker keratin 6a (KRT6A) and the basal marker KRT14. Additionally, interrogation of The Cancer Genome Atlas yielded a significant negative correlation between expression of *IGF1R* and *KRT6A* or *KRT14* in human breast tumors, providing the translational value of these findings to human disease.

## 2. Materials and Methods

### 2.1. Experimental Animals

All animal protocols were approved by the Rutgers University Institutional Animal Care and Use Committee (Newark, NJ, USA), and all experiments were managed in accordance with the NIH guidelines for the care and use of laboratory animals. Animal care was provided by the veterinary staff of the division of animal resources in the Rutgers Cancer Institute at University Hospital on the Rutgers Biomedical Health Sciences campus. The MMTV-*Wnt1* line on an FVB background [31] was obtained as a gift from Dr. Yi Li. The MMTV-*Wnt1*//MMTV-*dnIGF1R* (referred to here as DN-Wnt1) line was described previously [30]. All tumors were harvested once they reached 1.5 cm^3^.

### 2.2. Tissue Harvest, H&E, and Immunofluorescence

Freshly dissected tumors were washed with PBS, drop-fixed in 4% paraformaldehyde for 24 h at room temperature, and paraffin-embedded. Newly embedded tissues were sectioned at 5 µm thickness and stained as described previously [33]. In cases where a four-channel stain was performed, prior to mounting, the TrueVIEW Autofluorescence Quenching Kit (Vector Labs, SP840015, Newark, CA, USA) was used according to the manufacturer’s instructions. Mounting was performed by removing the slides from TBS, removing as much buffer as possible without disturbing the tissue, and adding ProLong Gold Antifade Mountant with DAPI (ThermoFisher, P36931, Waltham, MA, USA) or without (ThermoFisher, P36930), depending on whether or not a secondary antibody conjugated to a fluorophore excited in 350/405 range was used.

Primary antibodies used were rabbit anti-P63 (Abcam, ab124762, 1:400, Cambridge, England, UK), rat anti-KRT8 (DSHB, TROMA-I, 1:100, Iowa City, IA, USA), mouse anti-KRT14 (Invitrogen, MA5-11599, 1:100, Waltham, MA, USA), rabbit anti-KRT14 (Invitrogen, MA5-32214, 1:100), and rabbit anti-KRT6A (BioLegend, Cat# 905701, 1:500, San Diego, CA, USA). Secondary antibodies used were goat anti-rabbit-488 (Invitrogen, A11034, 1:750), goat anti-rat-546 (Invitrogen, A11081, 1:750), goat anti-mouse-647 (Invitrogen, A21235, 1:750). For all tumor immunofluorescent staining, a minimum of three biological replicates were stained per group, unless otherwise stated in the figure legend.

### 2.3. Brightfield and Immunofluorescent Microscopy

Brightfield imaging was performed on a Keyence BZ-X710 (Keyence, Osaka, Japan) at 4× magnification utilizing the Keyence imaging software (Keyence BZ-X Viewer v1.4.0.1). Image stitching was performed automatically by the software by manually indicating the top-left and bottom-right bounds of the tissue and enabling the stitch and auto-focus options, following a manual white balance set to an area devoid of tissue.

Fluorescent imaging was performed on a Keyence BZ-X710 (Keyence) at 20× or 60× magnification utilizing the Keyence imaging software (Keyence BZ-X Viewer v1.4.0.1). The correct plane within the tissue was found by using DAPI (or a reliable stain in the absence of DAPI) as a point of reference. The staining for each channel was manually black-balanced, focused, and imaged independently. Settings for each channel were kept consistent across samples. Each individual channel was dehazed using the same standardized settings and overlayed within the Keyence BZ-X Analyzer program (v1.4.1.1). Quantification of immunofluorescent images was performed for each stain by determining percent positivity per region of interest using the QuPath (v0.7) pixel classifier and normalized using negative controls [34].

### 2.4. cBioPortal Database Analysis

Analysis was performed on https://www.cbioportal.org by first selecting a cancer type, followed by a database of interest to be queried. The Cancer Genome Atlas (Firehose Legacy) database within the Invasive Breast Carcinoma section was selected due to its large sample size (1108 patients) and its availability of mRNA sequencing data. The database was queried for IGF1R and configured to include and display mRNA expression data by Z-score relative to all samples. IGF1R^High^ and IGF1R^Low^ groups were then created by narrowing the available patient set to only include samples that express higher than average IGF1R (z-score > 1) and lower than average IGF1R (z-score < −1). The de-identified numbers for patients in these cohorts were organized into groups through the cBioPortal’s create group function. These groups were then compared on a per gene basis for targets of interest using the built-in online comparison tools. The raw comparison data were then downloaded and processed with GraphPad Prism 9 to generate graphs. PAM50 subtype and survival analyses were performed as described above using the Molecular Taxonomy of Breast Cancer International Consortium database due to the lack of this information in the TCGA dataset [35].

### 2.5. Tumor Dissociation for scRNAseq

Animals used in this experiment were latency matched in the range of 6–8 weeks at the age of first tumor palpation. Dissected tumors were dissociated as described previously [32]. The cells were then spun for 3 min at 200 rcf to form a soft pellet. The supernatant was carefully discarded, and the pellets were washed by being thoroughly resuspended in 20 mL of HBSS (Gibco, 14175-095, Waltham, MA, USA). The tubes were spun again for 3 min at 200 rcf, and the supernatant was discarded. The samples were then resuspended in 10 mL of HBSS and filtered into new tubes through a 70 µm cell strainer (Falcon, 352350, Brookings, SD, USA). The original 50 mL tubes were washed out with an additional 10 mL of HBSS, which was then filtered into their respective tubes. Next, the samples were filtered into new tubes through a 40 µm cell strainer (Falcon, 352340), 10 mL at a time. A final 10 mL of HBSS was added to the 70 µm tubes to wash any remaining cells, and filtered through their appropriate 40 µm strainers. The samples were spun down a final time for 3 min at 200 rcf, and the supernatant was discarded. The pellets, composed mostly of single cells, were resuspended in 1 mL of HBSS and viable cells were counted using the trypan blue (Mediatech, 25-900-Cl, Hsinchu, Taiwan) exclusion method on a hemacytometer (Fisher Scientific, 0267110, Pittaburgh, PA, USA). Finally, the cells were moved to 1.5 mL tubes (USA Scientific, 1615-5500, Ocala, FL, USA) at a density of 80,000 cells/200 µL of 0.5% BSA/PBS, placed on ice, and delivered to the Genomics Core for further processing.

### 2.6. Single-Cell RNA Sequencing

Single cells were captured using the 10× Chromium system (10× Genomics, Pleasanton, CA, USA) and sequenced with the NovaSeq 6000 (Illumina, San Diego, CA, USA). Raw reads were barcode deconvoluted and aligned to the reference genome (mm10) via Cell Ranger (v7.0.1). All subsequent processing was performed using the Seurat package within R (v4.3.0). Ambient RNA was reduced (SoupX v1.6.2), low quality cells (cells with percentage of reads of mitochondrial origin >10%, with percentage of reads of ribosomal origin >45%, with <1000 feature counts, with >7000 feature counts) were filtered from the dataset, putative doublets were scrubbed assuming 10% multiplet rate (DoubletFinder v2.0.3), and read counts were normalized using the scTransform method. Samples were integrated with the Seurat integrate function and clustered via UMAP according to nearest neighbors. Following quality control filtering based on mitochondrial and ribosomal gene exclusion, the sample set was reduced to an average of 2924 eWnt1 cells/sample and 2720 eDN-Wnt1 cells/sample. Re-clustering was performed as above on subset clusters based on common annotation types.

### 2.7. Cell–Cell Interactions

Cell–cell interaction analysis was performed using LIANA (v0.1.11) in R (v4.3.0). Ligand–receptor interactions were inferred by combining predictions from multiple algorithms (including CellPhoneDB, CellChat, etc.) to yield a consensus score. Each sender-receiver pair was rank-scored for prioritization, and interactions were filtered based on adjusted *p*-value.

### 2.8. Ingenuity Pathway Analysis

A list of differentially expressed genes identified from pseudobulk RNA-seq analysis for the combined eWnt1 and eDN-Wnt1 epithelial clusters E3, E4, and E6 was uploaded to the Ingenuity Pathway Analysis client (Qiagen, Venlo, The Netherlands), which was used to generate a graphical summary of differentially regulated pathways and related molecules that characterize each cluster.

### 2.9. Data Processing and Statistical Analyses

All graphical data were expressed as the mean ± standard error. Statistical comparisons were carried out by Prism 9 software (GraphPad, Boston, MA, USA). The unpaired Student’s and Welch’s *t*-test were used for two-group comparisons where indicated. One-way analysis of variance using Benjamini–Hochberg correction or two-way analysis of variance using Dunnett’s post hoc tests were used for multiple treatment comparisons. All experiments were performed with a minimum of *n* = 3.

## 3. Results

### 3.1. eWnt1 and eDN-Wnt1 Mammary Tumors Have Similar Histopathological Characteristics and Cytokeratin Profiles

The MMTV-*Wnt1* transgenic mouse line has been utilized by the scientific community for over three decades as a translational model of human basal-like breast cancer, where Wnt signaling is often enriched and predictive of worse prognosis [20,22]. In 2019, Pfefferle et al. reported the existence of two distinct tumor phenotypes (denoted as Wnt1-Early^Ex^ and Wnt1-Late^Ex^) that arise in the MMTV-*Wnt1* line, distinguished by a latency cutoff of ~15 weeks of age [25]. In contrast to Wnt1-Late^Ex^, Wnt1-Early^Ex^ tumors had dramatically different histological features, an enhanced canonical Wnt pathway signature, and a significantly higher CD49F^+^/EPCAM^−^ epithelial population, which is consistent with the flow cytometry profile of normal human mammary stem cells [25,36]. Previously, we reported that inhibition of the IGF1R in the MMTV-*Wnt1* model through expression of a human, dominant-negative IGF1R transgene (MMTV-*Wnt1*/*dnIGF1R*) resulted in changes to tumor latency, the microenvironment, and adhesion, and also increased metastatic potential [30,31,32]. However, tumors were analyzed in these experiments prior to the Pfefferle report; they were not stratified or compared by latency. Consequently, we deemed it necessary to exclusively investigate the phenotypic differences between early Wnt1 (eWnt1) and early DN-Wnt1 (eDN-Wnt1) tumors, using the 15-week cutoff established by Pfefferle et al. [25].

**Figure 1:**
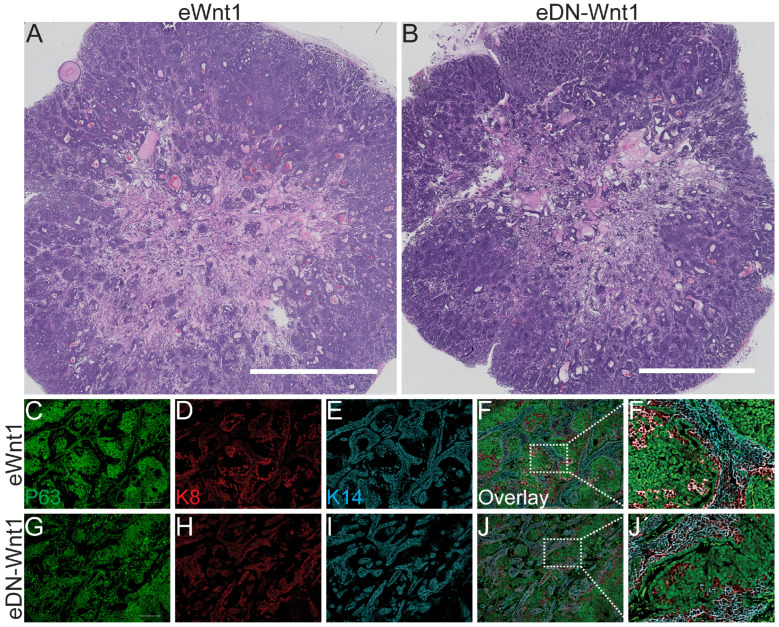
eWnt1 and eDN-Wnt1 tumors have similar gross histological features and epithelial marker expression. (**A**,**B**) Representative images of hematoxylin and eosin-stained tumor sections from eWnt1 and eDN-Wnt1 tumors, *n* = 3/group, scalebar = 5 mm. (**C**–**J**) Representative P63 (green), KRT8 (K8, red), and KRT14 (K14, cyan) immunofluorescent staining of eWnt1 (**C**–**F**) and eDN-Wnt1 (**G**–**J**) tumor epithelial cells. (**F’**,**J’**) Zoomed in fields of view taken as indicated from (**F**) and (**J**), respectively, *n* = 3/group, scalebars in (**C**) and (**G**) = 200 μm, and applied to (**C**–**F**) and (**G**–**J**).

Due to the major histological differences observed between early and late Wnt1 tumors, we performed hematoxylin and eosin staining to elucidate any potential macroscopic changes to eWnt1 tumors when IGF1R was inhibited. Early Wnt1 (Figure 1A) and eDN-Wnt1 (Figure 1B) appeared similar in their tissue architecture (*n* = 3/group). Both groups contained a loose inner core and exhibited dense epithelial clustering interspersed by long tracks of lightly staining extracellular matrix, consistent with the original histological description of early Wnt1 tumors [25]. Additionally, these dense epithelial clusters are characteristic of eWnt1 tumors and have been shown to lack expression of KRT5/keratin 8 (KRT8)/KRT14/keratin 18 (KRT18), while expressing both tumor protein 63 (P63) and G-protein coupled receptor 125 (GPR125, encoded by *Adgra3*), a marker of mammary progenitor cells [25,26]. In order to validate the phenotype of the eWnt1 tumors and determine any potential differences in cell populations within the eDN-Wnt1 tumors, we performed immunofluorescent staining for P63 (green), KRT8 (red), and KRT14 (cyan) (Figure 1C–J). The eWnt1 tumors stained positively for KRT8/KRT14 primarily between the clusters expressing P63 (Figure 1C–F), reproducing the observations from previous studies [25,26]. Interestingly, there appeared to be three discrete populations of epithelial cells that were either KRT8^High^/KRT14^High^, KRT8^High^/KRT14^Low^, or KRT8^Low^/KRT14^High^ (Figure 1F). Inhibition of IGF1R through confirmed expression of the dominant-negative IGF1R did not alter either the staining patterns or intensity of these epithelial targets in eDN-Wnt1 tumors, suggesting loss of IGF1R signaling did not alter gross histology of the eWnt1 tumors (Figure 1J, Supplemental Figure S1).

### 3.2. Single-Cell RNA Sequencing of eWnt1 and eDN-Wnt1 Tumors Reveals Previously Unidentified, Distinct Cell Populations

**Figure 2:**
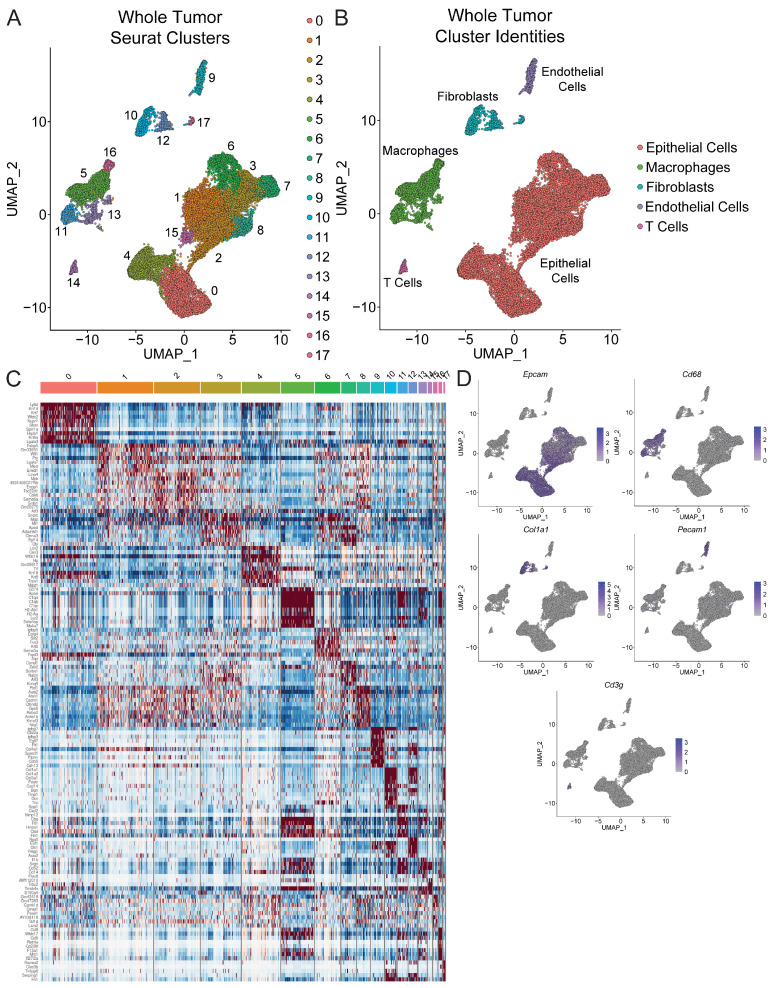
Single-cell RNA sequencing of eWnt1 and eDN-Wnt1 tumors reveals previously unidentified, distinct clusters. (**A**,**B**) Combined UMAP Seurat clustering (**A**) and identity annotations (**B**) of dissociated eWnt1 and eDN-Wnt1 tumors, *n* = 4/group. (**C**,**D**) Heat map (**C**) and single-gene feature plots (**D**) of marker genes used to determine cluster identity.

Our immunofluorescent strategy described above failed to reveal any overt changes in the abundance of specific epithelial subpopulations due to loss of IGF1R signaling; however, this technique is inherently limited in the number of targets per experiment that can be simultaneously detected. As a result, the more nuanced differences between these epithelial populations can only be identified with a less biased approach, such as single-cell RNA-sequencing. To more comprehensively investigate the cellular changes that occurred as a result of IGF1R inhibition in Wnt1-driven tumorigenesis, we performed scRNAseq on dissociated eWnt1 and eDN-Wnt1 tumors. Following single-cell capture, barcoding, sequencing, and quality control, the resulting data from all samples were integrated and clustered using the Uniform Manifold Approximation and Projection (UMAP) method according to nearest neighbor. The resulting UMAP plot visualizes all cell populations present in both tumors (Figure 2A, *n* = 4/group).

Eighteen different clusters (0–17) were identified that revealed transcriptionally distinct populations of cells (Figure 2A,B). Marker genes representing individual clusters were analyzed to determine the identity of each (Figure 2C,D). Unsurprisingly, the largest group, comprising clusters 0, 1, 2, 3, 4, 6, 7, 8, and 15, was epithelial cells distinguished by expression of *Epcam* (Figure 2B,D, peach). The next largest group, composed of clusters 5, 11, 13, and 16, was macrophages demarcated by expression of cluster of differentiation 68 (*Cd68*) (Figure 2B,D, green). Clusters 10, 12, and 17 were identified as fibroblasts by their expression of collagen, type 1, alpha 1 (*Col1a1*) (Figure 2B,D, blue). Clusters 9 and 14 were endothelial cells expressing cluster of differentiation 31/platelet endothelial cell adhesion molecule 1 (*Pecam1*) and T cells expressing cluster of differentiation 3 gamma (*Cd3g*), respectively (Figure 2B,D, purple, pink). These data used single-cell resolution to delineate novel, transcriptionally distinct cell populations that arise within eWnt1 tumors.

Additionally, we generated a gene expression heatmap to visualize the top marker genes characterizing each cluster (Figure 2C). Notably, cluster 0 (which denotes the largest cluster by cell abundance) was epithelial with *Krt14* as a top marker gene, in addition to high expression of *Krt8* (Figure 2C), revealing these cells as the presumed KRT8^+^/KRT14^+^ population previously identified by immunostaining in the primary tumors of both genotypes (Figure 1F,J).

### 3.3. Inhibition of IGF1R Alters Cell–Cell Interactions and the Transcriptomic Profile of Epithelial Subpopulations

**Figure 3:**
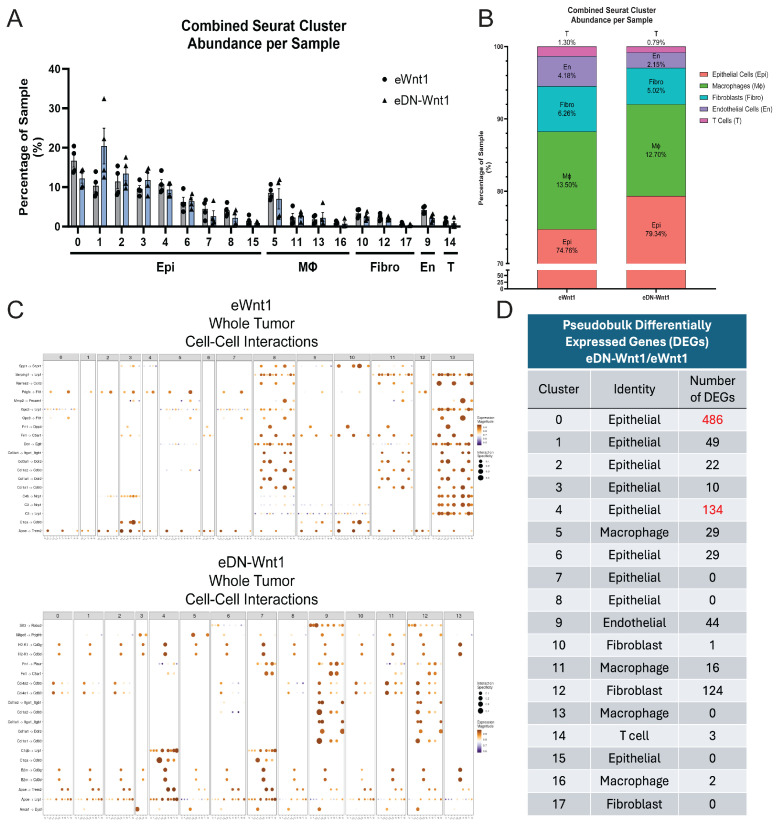
Reduction in IGF1R signaling induces changes to immune–stromal interactions and epithelial transcriptional profiles. (**A**,**B**) Quantification of cells per cluster as a percentage of total cells in each sample. (**C**) Cell–cell top interactions generated from genotype-specific scRNAseq clustering analysis of ligand/receptor pairs for eWnt1 (top) and eDN-Wnt1 (bottom) tumors. (**D**) Combined pseudobulk analysis of differentially expressed genes across all identified populations.

Once cluster identities were determined, we endeavored to explore macroscopic differences in the phenotype of the tumors. Surprisingly, quantification of each cluster by genotype did not yield statistically significant differences in cell abundance (Figure 3A). This suggests that any observed phenotypic changes that result from inhibition of IGF1R are more subtle and not due to overt alterations in population heterogeneity. Utilizing a more holistic approach, the clusters were then binned into groups based on cell identity and compared as a percentage. We found a ~5% increase in the total number of epithelial cells within eDN-Wnt1 tumors (Figure 3B). In the eWnt1 model, this 5% was distributed relatively evenly across the macrophage, fibroblast, endothelial, and T cell populations. The biggest shift was seen in the endothelial population with ~2% more endothelial cells in eWnt1 tumors than eDN-Wnt1 tumors (Figure 3B, purple). Although these tumors are overwhelmingly epithelial in composition, approximately 20–25% of eWnt1 and eDN-Wnt1 tumors are a mix of stromal and immune cells (Figure 3B).

The role of the immune system and extracellular matrix remodeling by cancer-associated fibroblasts (CAFs) in regulating cancer has been well studied [37,38,39]. The IGF1R signaling axis in CAFs has also been implicated in enhancing the aggressiveness of various cancer types, as well as in modulating immune cell infiltration [31,40,41,42]. As a result, and using our scRNAseq data, we reclustered eWnt1 and eDN-Wnt1 tumors individually by genotype and performed cell–cell interaction analysis for each group, where gene expression data for known ligand–receptor pairs can be used to infer potential interactions. Interestingly, after reclustering and stratifying by genotype, we identified 14 unique clusters in each of the eWnt1 and eDN-Wnt1 samples, whereas the combined clustering produced 18 unique clusters (Figure 2A and Figure 3C). 

Early Wnt1 only clusters 8, 11, and 13 were annotated as CAFs due to expression of multiple collagen genes and represented the cells with the most significant interactions. These cells were predicted to engage in both paracrine and autocrine signaling with all other cell populations through multiple ligand–receptor pairings, such as matrix metalloproteinase 2 (*Mmp2*)-*Pecam1* (CAF-endothelial), fibronectin 1 (*Fn1*)-dipeptidyl peptidase *(Dpp4*) (CAF-epithelial), *Col1a1*-cluster of differentiation 93 (*Cd93*) (CAF-immune) and *Col1a1*-discoidin domain receptor tyrosine kinase 2 (*Ddr2*) (CAF-CAF), suggestive of ongoing microenvironment remodeling (Figure 3C, top). This observation was consistent in eDN-Wnt1 tumors, where similar fibroblast interactions with other cell populations were the most significant, indicated by clusters 9 and 12. However, eDN-Wnt1 clusters 4 and 7, the cells involved in the second most significant interactions, were identified as immune cells through expression of *Cd68* (Supplemental Figure S2). The top interactions in these cells included complement C1q B chain (*C1qb*)-low-density lipoprotein receptor-related protein 1 (*Lrp1*), complement C1q A chain (*C1qa*)-*Cd93*, and beta-2-Microglobulin (*B2m*)-*Cd3g,* which are involved with apoptotic cell clearing and immune activation (Figure 3C, bottom). Notably, T cell activating interactions, such as *B2m*-*Cd3g* and *B2M*-cluster of differentiation 3 delta (*Cd3d*), were observed between T cells (cluster 13 in the eDN-Wnt1 cohort) and every other cell population. These appear specific to eDN-Wnt1 tumors, supporting a role for IGF1R in immune modulation.

Next, we pooled the scRNAseq data to form a combined pseudobulk RNAseq dataset. Using this approach, we were able to quantify and compare the number of differentially expressed genes (DEGs) as a ratio of eDN-Wnt/eWnt1 expression. Unsurprisingly, likely due to epithelial-specific expression of the *dnIGF1R* driven by the epithelial-specific MMTV promoter [19,43,44], gene transcription was most altered in epithelial cell clusters 0 and 4 with 486 and 134 DEGs, respectively (Figure 3D). Consequently, our experimental strategy pivoted to exploring gene expression changes in the epithelial populations exclusively and eliminating the contributions of other cell types.

### 3.4. Combined Reclustering of eWnt1 and eDN-Wnt1 Epcam^+^ Cells Identifies Novel, Transcriptionally Discrete Epithelial Progenitor Populations

**Figure 4:**
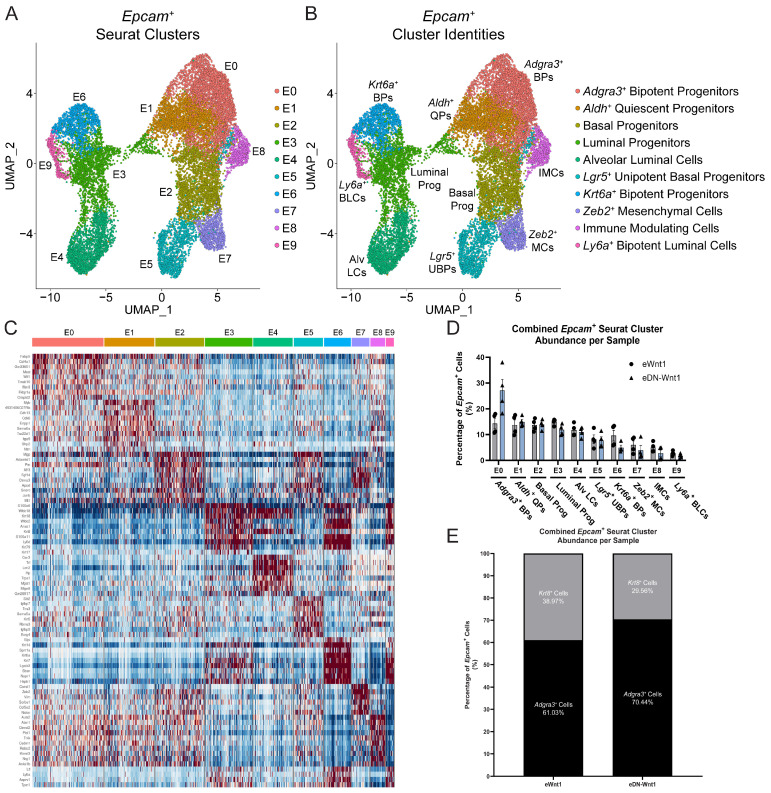
Combined reclustering of eWnt1 and eDN-Wnt1 *Epcam^+^* epithelial cells identifies novel *Adgra3^+^* progenitors. (**A**,**B**) Combined UMAP Seurat epithelial reclustering (denoted prefix “E”; (**A**)) and identity annotations (**B**) of *Epcam^+^* eWnt1 and eDN-Wnt1 epithelial cells, *n* = 4/group. (**C**) Heat map of marker genes used to determine cluster identity. (**D**) Quantification of cells per cluster as a percentage of total *Epcam^+^* cells in each sample. (**E**) Quantification of *Adgra3^+^* and *Krt8^+^* cells per cluster as a percentage of total *Epcam^+^* cells in each sample.

In addition to the fact that the phenotypes observed in eWnt1 and eDN-Wnt1 tumors are, at least initially, epithelial-driven, our scRNAseq analysis thus far also confirmed that *Epcam*^+^ epithelial cells compose ~70–80% of the entirety of the tumor (Figure 3B). As a result, we reclustered a subset of the whole tumor dataset based on the criterion of *Epcam* positivity in order to exclude the influence of the non-epithelial populations that could potentially be masking more nuanced differences between eWnt1 and eDN-Wnt1 epithelial cells. The subsequent UMAP plot generated through this strategy identified 10 transcriptionally distinct populations (Figure 4A,B). Marker gene lists were used to generate a heat map and identify relevant genes to determine cluster identity (Figure 4C). Utilizing this list and the large body of scientific literature investigating various populations of multipotent mammary gland epithelial cells during development, we annotated the reclustered tumor epithelial cells analogously to their developmental counterparts (Figure 4B, [45,46,47]).

Clusters E0, E1, E2, E5, E7, and E8 were positive for *Adgra3*/*Gpr125*, which was recently identified as a marker for a family of mammary progenitor cells that exhibit bipotency [26]. These clusters accounted for the majority of the epithelial cells within the tumors and constituted the first of two major groupings of cells identified in our analysis, presenting a more traditionally “basal-like” gene expression signature (Figure 4A, left). Cluster E0 expressed both *Adgra3* and tumor-related protein 63 (*Trp63*, P63), which would characterize them as bipotent progenitors as identified previously [26]. Cluster E1 was defined as aldehyde dehydrogenase (*Aldh^+^*) quiescent progenitors due to expression of aldehyde dehydrogenase 1 family, member A1 (*Aldh1a1*) and aldehyde dehydrogenase 1 family, member A1 (*Aldh1a3*) along with cyclin-dependent kinase inhibitor 2A (*Cdkn2a*), the gene encoding the cyclin-dependent kinase inhibitor P16. Expression of aldehyde dehydrogenases is known to be indicative of primitive, bipotent mammary epithelial cells [48,49]. Cluster E2 was characterized as basal progenitor cells due to expression of *Adgra3*, *Krt5*, secreted protein acidic and rich in cysteine (*Sparc*), and zinc finger E-box-binding homeobox 2 (*Zeb2*) [50]. Cluster E3 was the largest cluster of the second group of epithelial cells that appeared more “luminal-like” in their transcriptomic profiles and most closely resembled that of normal luminal progenitors through their expression of *Aldh1a3*, WAP four-disulfide core domain 18 (*Wfdc18*), *Krt8*, and *Krt18* [49,51]. Cluster E4 was designated as alveolar luminal cells indicated by expression of the milk gene casein 3 (*Csn3*), as well as *Krt8*, and *Krt18* and their proximity adjacent to the luminal progenitor cluster, E3 (Figure 4B, left).

Cluster E5 was denoted as leucine-rich repeat-containing G-protein couple receptor 5^+^ (*Lgr5^+^*) bipotent progenitors due to their expression of *Lgr5*, *Adgra3*, *Trp63*, *Aldh1a1*, and *Aldh1a3* [52,53]. These cells also expressed *Krt5*, a basal marker, conforming with the original observations of these cells existing as a subset of the basal population by flow cytometric analysis [52]. Cluster E6 were *Krt6a^+^* bipotent progenitors, most distinguished by their expression of *Krt6a* as a top marker gene, as well as *Krt8* and *Krt18*. *Krt6a* characterizes a bipotent mammary epithelial population in the normal gland [54]. Cluster E7 were *Zeb2^+^* mesenchymal cells as a result of their similarity to cells undergoing epithelial–mesenchymal transition, such as expression of *Zeb2* and vimentin (*Vim*), and lack of expression of cadherin 1 (*Cdh1*). Interestingly, *Zeb2^+^* cells in the normal mammary gland are a small subset of basal cells, having been described as “terminally mesenchymal” and lacking the capacity to reconstitute multiple epithelial populations upon transplantation [55]. Cluster E8 provided the most difficulty in annotation due to its lack of expression of previously described marker genes, however, a unifying ontology in the literature among marker genes including ataxin 1 (*Atxn1*), X-inactive specific transcript (*Xist*), par-3 family cell polarity regulator (*Pard3*), phospholipase C-like 1 (*Plcl1*), and cell adhesion molecule 1 (*Cadm1*) is immune modulator, hence we described this cluster as immune modulating epithelial cells [56,57,58,59,60]. The final, and smallest, cluster E9 was characterized by its expression of lymphocyte antigen 6 family member A/steam cell antigen 1 (*Ly6a*/*Sca1*). *Ly6a^+^* cells in the normal mammary gland are situated within the luminal compartment and possess biopotency upon transplantation into cleared mammary fat pads [61].

Similar to the analysis performed previously on the combined whole tumor clustering (Figure 3), we quantified the number of *Epcam^+^* epithelial cells comprising each cluster to explore potential population shifts between genotypes. No significant changes were observed in any cluster, although there was a trending increase in cluster E0 representing *Adgra3^+^* bipotent progenitors in eDN-Wnt1 tumors (Figure 4D). Mature, differentiated luminal cells of the mammary gland express *Krt8*. In these data, *Krt8* expression was limited to E3, E4, E6, and E9, whereas expression of the known progenitor marker *Adgra3* was exclusive to clusters E0, E1, E2, E5, E7, and E8 (Supplemental Figure S3). Quantification of two groups created using these criteria provides an indirect look at the balance of differentiation within these tumors. Tumors expressing the *dnIGF1R* contained ~9% more *Adgra3^+^* cells (Figure 4E). This observation supports the hypothesis posed by us previously that inhibition of IGF1R may disproportionately affect cells of the luminal lineage and compromise their capacity for differentiation [27].

### 3.5. Inhibition of IGF1R Signaling Disproportionately Affects Gene Transcription in “Luminal-like” Cells

**Figure 5:**
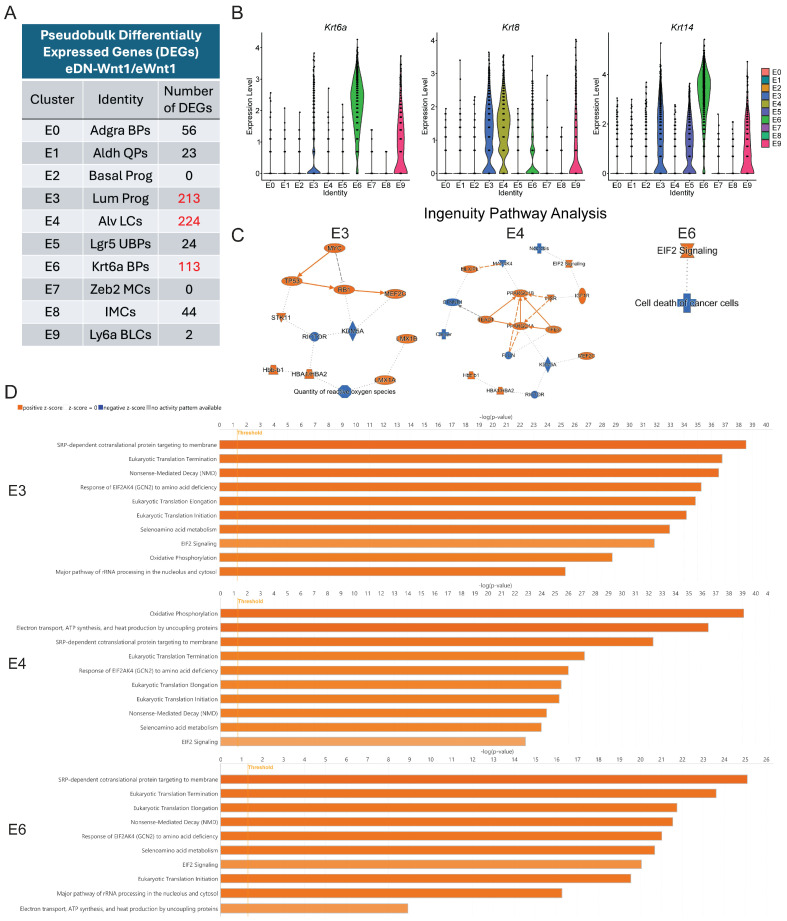
Epithelial-specific pseudobulk and Ingenuity Pathway Analysis of eDN-Wnt1 tumors. (**A**) Quantification of the number of differentially expressed genes in each cluster. (**B**) Violin plots depicting expression of *Krt6a*, *Krt8*, and *Krt14*. (**C**) IPA graphical summaries generated from scRNAseq data indicate the regulation of related molecules within clusters. (**D**) List of pathways predicted to be activated based on gene expression within clusters. Orange = predicated activation, blue = predicted inhibition.

In addition to modifications in cellular subpopulation heterogeneity, differential gene expression is another important contributor to deciphering any phenotypic changes downstream of IGF1R inhibition. To determine transcriptional alterations that take place within the identified epithelial subpopulations of eDN-Wnt1 tumors, we performed pseudobulk analysis between genotypes and within each epithelial cluster to quantify the number of differentially expressed genes (Figure 5A). Of the ten epithelial clusters, the basal progenitors (E2), *Zeb2^+^* mesenchymal cells (E7), and *Ly6a^+^* bipotent luminal cells had two or fewer significantly altered genes, suggesting a lack of contribution to the overall phenotype. The *Adgra3^+^* bipotent progenitors (E0) had 56, *Aldh^+^* quiescent progenitors (E1) had 23, *Lgr5^+^* bipotent progenitors (E5) had 24, and the immune modulating cells (E8) had 44 significantly altered genes. The clusters with the highest number of significant changes were the luminal progenitors (E3) with 213, alveolar luminal cells (E4) with 224, and *Krt6a^+^* bipotent progenitors (E6) with 113 altered genes, respectively (Figure 5A). It is noteworthy that the luminal progenitors and *Krt6a^+^* bipotent progenitors were the clusters responsible for the highest levels of *Krt14* expression (Figure 5B), which is an established marker for more invasive cancers [13,14].

Because these clusters had the largest number of significantly altered genes between genotypes, we performed Ingenuity Pathway Analysis (IPA) on the individual pseudobulk datasets for E3, E4, and E6 to identify any affected intracellular networks in tumors with inhibited IGF1R. Graphical summaries were generated to illustrate potential molecular interactions (Figure 5C). Cluster E3 (luminal progenitors) expression data suggested activation of the transcription factor P53 and Myc proto-oncogene (MYC, Figure 5C, left), both of which have been linked to a more aggressive breast cancer phenotype [62,63]. Interestingly, cluster E4 (alveolar luminal cells), the most differentiated epithelial population, was also the cluster with the most differentially expressed genes compared to the corresponding population in eWnt1 tumors. Two of the most significant hits were for activation of peroxisome proliferator-activated receptor gamma coactivator 1-alpha (*Ppargc1a*) and beta (*Ppargc1b*), which are two genes involved in metabolic homeostasis and mitochondrial function (Figure 5C, center). Hyperactivation of *Ppargc1a* in breast cancer results in enhanced oxidative phosphorylation and metastasis [64,65]. Importantly, insulin-like growth factor 1 (IGF1) signaling through IGF1R contributes to mitochondrial biogenesis, providing a potential link between expression of the dnIGF1R and the epithelial phenotype [66]. Furthermore, analysis of the *Krt6a+* cluster E6 identified eIF2 signaling activation and inhibition of cell death (Figure 5C, right). EIF2 signaling is upregulated during endoplasmic reticulum stress and was previously shown to be activated with pharmacological inhibition of IGF1R [31].

Common to all three clusters with the highest number of significantly altered genes, the top modified pathways with IGF1R inhibition were upregulation of oxidative phosphorylation and signal recognition particle (SRP) co-translational protein targeting to endoplasmic reticulum membrane pathways, as well as downregulation of mitochondrial dysfunction, suggesting a metabolic phenotype within these cells (Figure 5D). Multiple pathways involving translation initiation, elongation, and termination were predicted to be activated in all three clusters. Consistent with this, the IGF1R resides upstream of the mechanistic target of rapamycin–(mTOR)–protein kinase B (AKT) axis, which is a major regulator of protein translation. Together, these analyses further contribute to the growing body of evidence suggesting that IGF1R inhibition disproportionately affects cells of the luminal lineage in the eDN-Wnt1 model.

### 3.6. Krt6a Is Expressed by Cells in Metastatic Lesions of All Sizes and Upregulated in Human Patients with Low Expression of IGF1R

In the breast, expression of cytokeratins such as *Krt5*, *Krt6*, *Krt8*, *Krt14*, and *Krt18* correlates with epithelial differentiation status and tumor progression (for ref. [67]). *Krt6a* is expressed in the terminal end buds of proliferating mammary ductal outgrowths during development, suggesting a progenitor-like role for these cells [68]. Importantly, KRT6A^+^ epithelial cells have been previously identified in MMTV-*Wnt1* tumors and exhibit heightened canonical Wnt signaling [69]. In addition to its role in TNBC, *Krt6a* expression has been shown to positively contribute to the aggressiveness and metastatic capacity of other types of cancer, such as lung, bladder, and head and neck [70,71,72,73,74].

**Figure 6:**
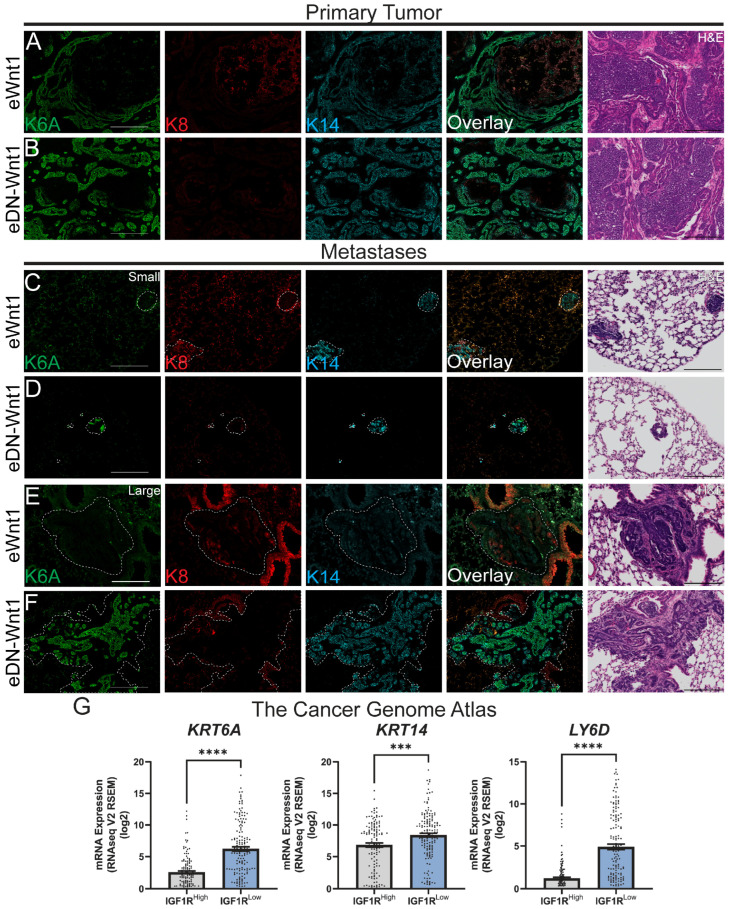
Downregulation of IGF1R signaling maintains the KRT6A^+^ population within lung metastases and negatively correlates with expression of keratins in human breast cancer patients. (**A**–**F**) Representative KRT6A (green), KRT8 (red), and KRT14 (cyan) immunofluorescent staining of eWnt1 (**A**) and eDN-Wnt1 (**B**) primary tumors and both small (**C**,**D**) and large (**E**,**F**) metastatic lesions from both genotypes with corresponding H&E, *n* = 3/group. All images are 20×. (**G**) cBioPortal mRNA sequencing analysis for select marker genes of clusters E3, E4, and E6 in The Cancer Genome Atlas (Firehose Legacy) human breast cancer database cohorts stratified by *IGF1R* expression, *n* = 128 and *n* = 155 for the IGF1R^High^ and IGF1R^Low^ cohorts respectively. *** = *p* ≤ 0.001, **** = *p* ≤ 0.0001.

Furthermore, our scRNAseq experiments have demonstrated that *Krt6a* expression denotes a specific subset of epithelial cells within the eWnt1 and eDN-Wnt1 tumors (Figure 4B) that also concomitantly express *Krt14*, implicating these cells in the potential seeding of metastases in these tumors. This population of cells also exhibited the third largest number of differentially expressed genes in our dataset (Figure 5A). In order to explore the expression of *Krt6a*, we performed additional immunofluorescent staining for KRT6A, KRT8, and KRT14 on eWnt1 and eDN-Wnt1 primary tumors (Figure 6A,B). Similarly to previous immunofluorescent experiments, there were no obvious differences in KRT6A staining intensity or number of KRT6A-expressing cells between genotypes (Figure 6A,B, green), which is consistent with our scRNAseq analysis that failed to find a significant difference in *Krt6a* expression in these cells. However, when we applied this staining strategy to metastatic lesions within eWnt1 and eDN-Wnt1 lungs, we found that the smallest clusters of tumor cells in eWnt1 lungs were weakly positive for KRT6A, whereas eDN-Wnt1 cells stained strongly for KRT6A (Figure 6C,D, green). In eDN-Wnt1 metastases, these KRT6A^+^ cells were also strongly double positive for KRT14, suggesting a more aggressive, stem-like phenotype. These cells also appeared to dominate eDN-Wnt1 metastases of larger sizes and were conspicuously more abundant than in eWnt1 metastases (Figure 6E,F). These data suggest that the bipotential KRT6A^+^ cells are possibly responsible for seeding metastases in both groups; however, the KRT6A^+^ population is expanded with the growth of the lesion in metastases with low IGF1R signaling.

To determine whether any correlation exists between *KRT6A* and/or *KRT14* with expression of *IGF1R* in human breast tumors, we utilized cBioPortal to explore this possibility within The Cancer Genome Atlas (Firehose Legacy) human breast cancer database. Critically, we previously demonstrated that patients with low levels of *IGF1R* expression have significantly increased lymph node positivity and overall survival when compared to patients with tumors expressing high levels of the receptor [27,31]. The new analysis revealed a statistically significant negative correlation between expression of *IGF1R* and both *KRT6A* and *KRT14* (Figure 6G, left, middle). A significant negative correlation was also found between *IGF1R* and lymphocyte antigen 6 family member D (*LY6D*) (Figure 6G, right), a well-known gene whose expression has been shown to correlate with increased metastasis and poorer prognosis in many different cancer types [75]. Importantly, *Ly6d* is the top marker gene denoting the *Krt6a^+^* population in our scRNAseq analysis (Figure 4C). In addition, subsequent binning of patients based on both *IGF1R* expression level and PAM50 subtype was performed on the Molecular Taxonomy of Breast Cancer International Consortium dataset (Supplemental Figure S4). These data demonstrate an apparent relationship between low *IGF1R* expression and a BLBC diagnosis (Supplemental Figure S4A,B). Patients in the IGF1R^Low^ group also had worse overall and relapse-free survival compared to IGF1R^High^, strengthening the correlation between *IGF1R*, *KRT6A*, *KRT14* and patient prognosis ([31], Supplemental Figure S4C,D).

In conclusion, these data suggest that inhibition of IGF1R in eWnt1-driven mammary tumors induces transcriptional changes that enhance the metastatic capacity of KRT6A^+^ epithelial cells and identify *Krt6a* as a potential biomarker for metastatic breast cancer.

## 4. Discussion

Our data reveal a previously underappreciated relationship between the IGF1R, canonical Wnt signaling, and transcriptional regulation of the greater tumor phenotype during disease progression. Here, we have demonstrated a role for IGF1R in the differentiation of mammary epithelial cells during early Wnt-driven tumorigenesis. These alterations, as a result of IGF1R inhibition, uncovered macroscopic changes in cell–cell interactions, as well as identified a number of transcriptionally distinct epithelial subpopulations. Some of these markers, such as *KRT6A* and *KRT14*, negatively correlated with *IGF1R* expression in humans, suggesting mechanistic conservation and supporting the translational value of these targets across species.

Cytokeratins are intermediate filament proteins that provide structure and resistance against mechanical stress to the cell. Additionally, their expression has been utilized to infer epithelial differentiation states, as well as to characterize tumor phenotypes and monitor patients for metastatic spread to non-epithelial tissues [67]. However, the exact molecular mechanisms through which cytokeratins contribute to metastasis are less clearly understood. Of particular interest, KRT14 has been shown to be critical for invasion and metastasis in the highly metastatic MMTV-*PyMT* mouse mammary tumor line [13]. In this model, knockdown of KRT14 was sufficient to abrogate invasion and metastasis of these cells both *in vitro* and *in vivo*, suggesting KRT14 potentially provides the structural plasticity required to invade and migrate through the extracellular matrix. Expression of *Krt14* suggests it may be playing a role in both the primary tumor phenotype and metastatic lesions that form from tumors with inhibited IGF1R (Figure 1 and Figure 6).

In addition to KRT14, our work further supports the importance of another keratin, KRT6A (Figure 6). KRT6A identifies a bipotent mammary progenitor cell in the developing mammary gland, and its expression has been shown to have tumor-promoting effects in a number of different cancer models, much like KRT14 [68,69,76]. Positive expression of *KRT6A* in metastatic lesions resulting from tumors with inhibited IGF1R suggests the possibility that these cells are responsible for metastasis in our model. However, this interpretation is complicated by the observation that the majority of cells expressing KRT6A (traditionally a luminal marker) are also double positive for KRT14 (a traditionally basal marker). This contrasts with earlier developmental studies on KRT6A that found isolated KRT6A^+^ cells were KRT8^−^ and emphasizes the dysregulation of cytokeratin expression that occurs during tumorigenesis. Interestingly, this same study induced transformation in KRT6A^+^ cells through expression of constitutively activated Harvey rat sarcoma viral oncogene homolog (*Hras*) and the resulting tumors exhibited similar histopathological features to our early tumors, such as containing clusters of epithelial cells surrounded by dense stroma and dysregulation of *Krt6* and *Krt8* expression, which are characteristic of early Wnt1 tumors [77].

There is an expanding literature that demonstrates lineage plasticity in breast tumors where cells that appear phenotypically luminal gain expression of basal markers [16,78]. Importantly, the IGF1R is known to be involved in epithelial differentiation of the normal mammary gland [29]. Thus, inhibition of the receptor in the Wnt1 tumors could feasibly result in aberrant differentiation of luminal cells and cause them to gain basal characteristics. This hypothesis is supported by our scRNAseq data, where the clusters that appear the most “luminal-like” are responsible for the most differentially expressed genes (Figure 5). Moreover, the hypothesis that the luminal clusters in our dataset are gaining basal characteristics is bolstered by the fact that if KRT14 was expressed and maintained early and throughout tumorigenesis, we would expect to observe its expression in most, if not all, of the epithelial clusters. However, this is not the case, and *Krt14* is only expressed in four out of the ten combined epithelial clusters (Figure 5B).

The heterogeneous nature of basal-like breast cancer highlights the utility of scRNAseq as a tool to more clearly differentiate between different tumor models classified within this molecular subtype. For example, scRNAseq of MMTV-*PyMT* tumors, which are classified as basal-like, revealed eight distinct epithelial populations, where seven of these appeared phenotypically luminal while one appeared basal [79]. However, in this report, the authors classified “luminal cells” based on their expression of KRT8 and KRT18, while it has been demonstrated elsewhere that KRT8-expressing cells within MMTV-*PyMT* tumors gain expression of KRT14, which they used as a marker of basal cells [13,79]. Both our immunofluorescent and scRNAseq data support the presence of these double-positive KRT8/KRT14 in both the primary tumors and metastases of both our models, drawing a similarity between MMTV-*PyMT* and early Wnt1-driven tumors (Figure 1 and Figure 6). Additionally, Cheung et al. showed the presence of KRT8/KRT14 double-positive cells within the smallest observed metastases, consistent with our data (Figure 6, [13]). Another mouse mammary tumor model utilizing beta-lactoglobulin (Blg)-Cre recombinase *(Cre*) to knockout breast cancer type 1 susceptibility protein (*BRCA1*) specifically in the luminal lineage showed that the resulting tumors were basal-like and resembled human *BRCA1*-driven tumors [78]. These authors concluded that the cell of origin for these tumors was a luminal progenitor, and further interrogation of their dataset confirmed the expression of KRT14 in at least some of these tumors, a characteristic linking these otherwise unique models of basal-like breast cancer [78]. Taken together, these data suggest an important role for these double-positive cells in tumorigenesis and the metastatic process that is common across multiple models of metastatic breast cancer.

Our earlier reports on the MMTV-*dnIGF1R* and MMTV-*Wnt1* tumor models, as well as a luminal lineage-specific deletion of the *Igf1r* in MMTV-*Wnt1* tumors, indicated that increased lung metastases are associated with decreased IGF1R signaling or *Igf1r* deletion ([30,32]). However, these studies were done without stratifying by early or late Wnt1 tumor phenotype. Since the early tumors initiate prior to ~12 weeks of age, the number and size of metastases are low. We now have data where we specifically determined metastatic rates in early vs. late MMTV-*Wnt1* tumors plus or minus lineage-specific *Igf1r* deletion. These data indicate that the early Wnt tumors have a low metastatic rate, and loss of the *Igf1r* in these early tumors has no impact on lung metastases. In contrast, loss of the receptor in late Wnt1 tumors significantly increases the number of lung metastases and often results in larger metastases [80]. Our current findings in the early tumors identify triple-positive (KRT14/KRT8/KRT6A) cells in the tumors, which are prominent in the smallest lung metastases we detected and whose presence is maintained as metastases grow (Figure 6D,F). Thus, our interpretation is that loss of IGF1R signaling enhances this population such that with increased tumor latency, they are more likely to either seed metastases and/or expand once seeded in the lung. This interpretation is also supported by the human data shown here and in our prior reports, indicating that low *IGF1R* correlates with lower survival and with increased expression of *KRT6A*/*KRT14*.

The role of the IGF1R in breast tumorigenesis remains complex. This is compounded by multiple reports in the literature demonstrating that both constitutive activation and inhibition of IGF1R in the mammary gland result in an increased luminal progenitor population [29,30]. Due to the consistencies between the eDN-Wnt1 tumors and other models of basal-like breast cancer discussed above, it is feasible to hypothesize that the cell of origin in our tumors is a luminal progenitor; however, this remains to be empirically determined and is complicated by the observation that tumors initiated in a KRT6A^+^ cell contain similar histological features to both eWnt1 and eDN-Wnt1 tumors [77]. This report identifies high expression of a keratin, KRT6A, that is expressed in KRT14^+^ cells present in both the primary tumor and metastatic lesions when IGF1R is inhibited in the MMTV-*Wnt1* tumor model. Expression of *Krt6a* also marks an early, stem-like epithelial progenitor population present during normal mammary development [54]. Furthermore, eWnt1 tumors also express an undifferentiated, stem-like phenotype, which could contain cells closely analogous to those present in the developing gland [25,26]. Thus, inhibition of IGF1R could interfere with differentiation, resulting in a population of cells that exhibit a hybrid luminal-basal phenotype denoted by expression of KRT8, KRT14, and KRT6A. As a result, expression of *Krt6a* could potentially be used as a biomarker for the metastatic potential of basal-like breast cancers.

## 5. Conclusions

We have provided evidence that inhibition of IGF1R in Wnt1-driven basal-like breast cancer results in the generation of metastatic cells that display phenotypic similarities corresponding to that of this less differentiated luminal population. This population expresses *Krt6a* and *Krt14*, which could potentially be used as biomarkers for metastatic basal-like breast cancer, especially in patients whose tumors are poorly differentiated.

## Data Availability

The original data presented in this study will be openly available at the time of publication. The names of the repository/repositories and accession number(s) will be included upon manuscript publication.

## Supplementary Materials

The following supporting information can be downloaded at: https://www.mdpi.com/article/10.3390/cancers18111749/s1, Figure S1. Analysis of differentiation markers and dnIGF1R expression in early tumors. (A–C), Immunofluorescent quantification of KRT8 (A), KRT14 (B), and P63 (C) in early tumors with or without dnIGF1R expression (*n* = 3/group). D, Quantitative PCR data confirming the expression of the human dnIGF1R in early tumor cells (*n* = 4/group). Welch’s *t*-test was used to determine significance indicated by *p* < 0.05. Figure S2. Identification of immune cells within the eDN-Wnt1 scRNAseq dataset. A, Whole tumor UMAP Seurat clustering of eDN-Wnt1 tumors, *n* = 4. B, Expression feature plot for the pan-immune marker, *Cd68*. Figure S3. Epithelial differentiation marker expression in the combined clustering *Epcam*^+^ tumor cell dataset. (A–C), Violin plots depicting cluster specific expression of *Adgra3* (A), *Aldh1a1* (B), and *Aldh1a3* (C). Figure S4. cBioPortal analysis of clinical characteristics associated with low expression of *IGF1R* in the METABRIC human breast cancer dataset. (A,B), mRNA sequencing analysis for *KRT6A* (A) and *KRT14* (B) in IGF1R^High^ (left) vs IGF1R^Low^ (right) patients stratified by PAM50 subtype. Basal *n* = 3, LumA *n* = 149, LumB *n* = 124 for IGF1R^High^ and Basal *n* = 113, LumA *n* = 24, LumB *n* = 25 IGF1R^Low^ cohorts respectively. *p*-values represent one-way ANOVA and q-values represent Benjamini-Hotchberg corrections with significance denoted as *p* < 0.05. (C,D), Kaplan-Meier curve indicating the overall (C) and relapse-free (D) survival of IGF1R^High^ (blue) vs IGF1R^Low^ (red) cohorts. IGF1R^High^, *n* = 300 and IGF1R^Low^, *n* = 415. Statistical significance was determined by Log-rank test and *p*-values < 0.05 were considered significant.

## Abbreviations

The following abbreviations are used in this manuscript:

ADGRA3	Adhesion G-protein-coupled receptor A3
AKT	Protein kinase B
ALDH	Aldehyde dehydrogenase
ALDH1A1	Aldehyde dehydrogenase 1 family, member A1
ALDH1A3	Aldehyde dehydrogenase 1 family, member A3
ATXN1	Ataxin 1
B2M	Beta-2-Microglobulin
BLBC	Basal-like breast cancer
BLG	Beta-lactoglobulin
BRCA1	Breast cancer type 1 susceptibility protein
C1QA	Complement C1q A chain
C1QB	Complement C1q B chain
CADM1	Cell adhesion molecule 1
CAF	Cancer-associated fibroblast
CD3D	Cluster of differentiation 3 delta
CD3G	Cluster of differentiation 3 gamma
CD49F	Cluster of differentiation 49F
CD68	Cluster of differentiation 68
CD93	Cluster of differentiation 93
CDH1	Cadherin 1
CDKN2A	Cyclin-dependent kinase inhibitor 2A
CDNA	Complementary deoxyribonucleic acid
COL1A1	Collagen, type 1, alpha 1
CRE	Cre recombinase
CSN3	Casein 3
DDR2	Discoidin domain receptor tyrosine kinase 2
DEG	Differentially expressed gene
DPP4	Dipeptidyl peptidase
eDN-Wnt1	Early dominant-negative-Wnt1
eIF2	Eukaryotic initiation factor 2
EpCAM	Epithelial cell adhesion molecule
ER	Estrogen receptor
eWnt1	Early Wnt1
FN1	Fibronectin 1
GPR125	G-protein coupled receptor 125
HER2	Erb-B2 receptor tyrosine kinase 2
HRAS	Harvey rat sarcoma viral oncogene homolog
IGF1	Insulin-like growth factor 1
IGF1R	Insulin-like growth factor 1 receptor
IPA	Ingenuity pathway analysis
KRT14	Keratin 14
KRT18	Keratin 18
KRT5	Keratin 5
KRT6A	Keratin 6A
KRT8	Keratin 8
LGR5	Leucine-rich repeat-containing G-protein couple receptor 5
LRP1	Low density lipoprotein receptor-related protein 1
LY6A	Lymphocyte antigen 6 family member A
LY6D	Lymphocyte antigen 6 family member D
MMP2	Matrix metalloproteinase 2
MMTV	Mouse mammary tumor virus
mTOR	Mechanistic target of rapamycin
MYC	Myc proto-oncogene
p63	Tumor protein 63
PARD3	Par-3 family cell polarity regulator
PECAM1	Platelet endothelial cell adhesion molecule 1
PLCL1	Phospholipase C-like 1
PPARGC1A	Peroxisome proliferator-activated receptor gamma coactivator 1-alpha
PPARGC1B	Peroxisome proliferator-activated receptor gamma coactivator 1-beta
PyMT	Polyoma middle t
RNA	Ribonucleic acid
RNAseq	Ribonucleic acid sequencing
SCA1	Stem cell antigen 1
scRNAseq	Single-cell ribonucleic acid sequencing
SPARC	Secreted protein acidic and rich in cysteine
SRP	Signal recognition particle
TNBC	Triple negative breast cancer
TRP63	Tumor related protein 63
UMAP	Uniform Manifold Approximation and Projection
VIM	Vimentin
WFDC18	WAP four-disulfide core domain 18
WNT1	Wnt family member 1
XIST	X-inactive specific transcript
ZEB2	Zinc finger E-box-binding homeobox 2
