# Supplementary material for: Inhibition of IGF1R in Early MMTV-Wnt1 Mammary Tumors: A Transcriptomic Analysis
Source: Cancers (Basel). 2026 May 27;18(11):1749. doi: 10.3390/cancers18111749 (PMC13256066; doi:10.3390/cancers18111749)
Supplement: Supplementary file 1 [file cancers-18-01749-s001.zip › Final_Supplemental Figure S1.pdf]

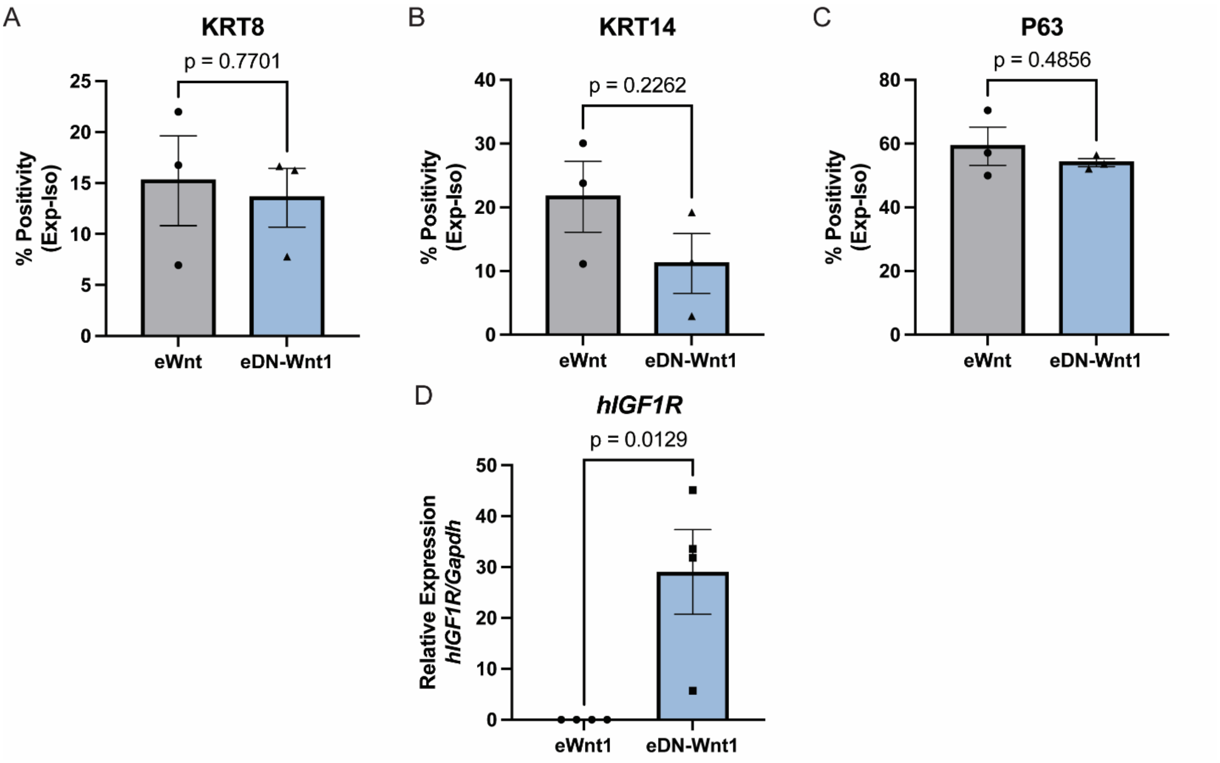

Supplemental Figure S1. Analysis of differentiation markers and *dnIGF1R* expression in early tumors. **A-C**, Immunofluorescent quantification of KRT8 (A), KRT14 (B), and P63 (C) in early tumors with or without *dnIGF1R* expression (n = 3/group). **D**, Quantitative PCR data confirming the expression of the human *dnIGF1R* in early tumor cells (n = 4/group). Welch's t-test was used to determine significance indicated by $p < 0.05$.
