# Supplementary material for: Inhibition of IGF1R in Early MMTV-Wnt1 Mammary Tumors: A Transcriptomic Analysis
Source: Cancers (Basel). 2026 May 27;18(11):1749. doi: 10.3390/cancers18111749 (PMC13256066; doi:10.3390/cancers18111749)
Supplement: Supplementary file 1 [file cancers-18-01749-s001.zip › Final_Supplemental Figure S2.pdf]

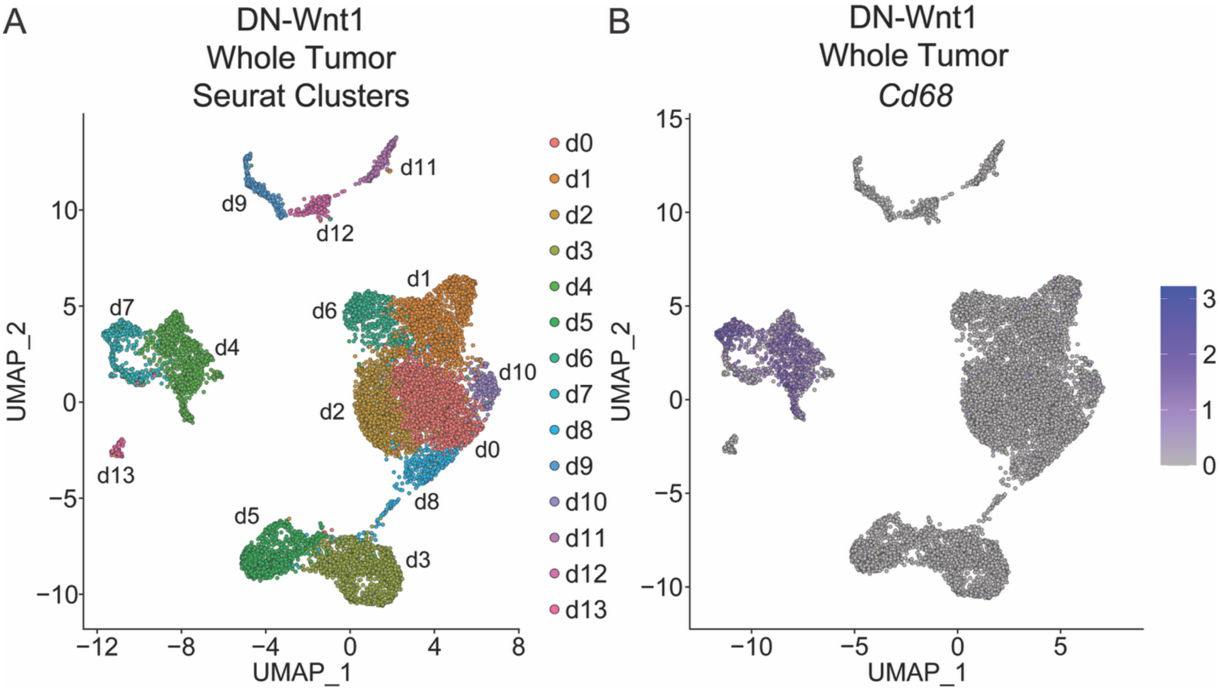

Supplemental Figure S2. Identification of immune cells within the eDN-Wnt1 scRNAseq dataset. **A**, Whole tumor UMAP Seurat clustering of eDN-Wnt1 tumors, n = 4. **B**, Expression feature plot for the pan-immune marker, *Cd68*.
