# Supplementary material for: Inhibition of IGF1R in Early MMTV-Wnt1 Mammary Tumors: A Transcriptomic Analysis
Source: Cancers (Basel). 2026 May 27;18(11):1749. doi: 10.3390/cancers18111749 (PMC13256066; doi:10.3390/cancers18111749)
Supplement: Supplementary file 1 [file cancers-18-01749-s001.zip › Final_Supplemental Figure S3.pdf]

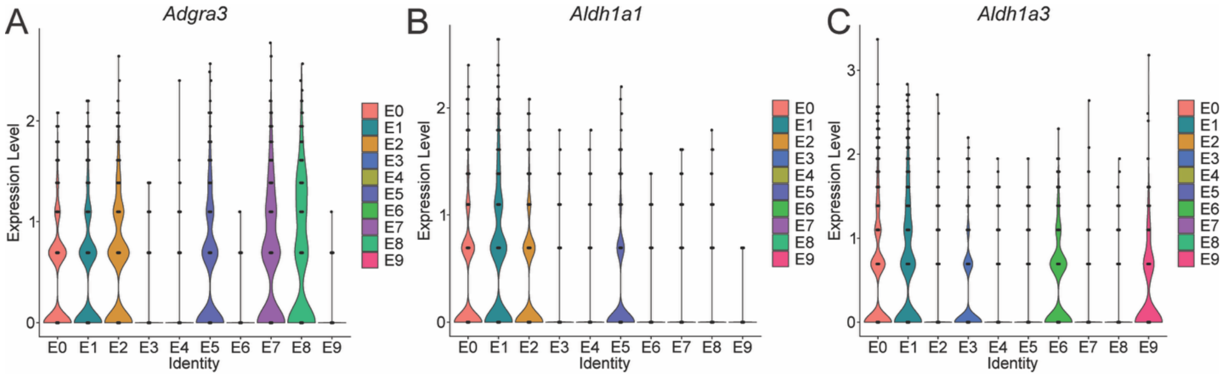

Supplemental Figure S3. Epithelial differentiation marker expression in the combined clustering *Epcam*+ tumor cell dataset. **A-C**, Violin plots depicting cluster specific expression of *Adgra3* (A), *Aldh1a1* (B), and *Aldh1a3* (C).
