# Supplementary material for: Inhibition of IGF1R in Early MMTV-Wnt1 Mammary Tumors: A Transcriptomic Analysis
Source: Cancers (Basel). 2026 May 27;18(11):1749. doi: 10.3390/cancers18111749 (PMC13256066; doi:10.3390/cancers18111749)
Supplement: Supplementary file 1 [file cancers-18-01749-s001.zip › Final_Supplemental Figure S4.pdf]

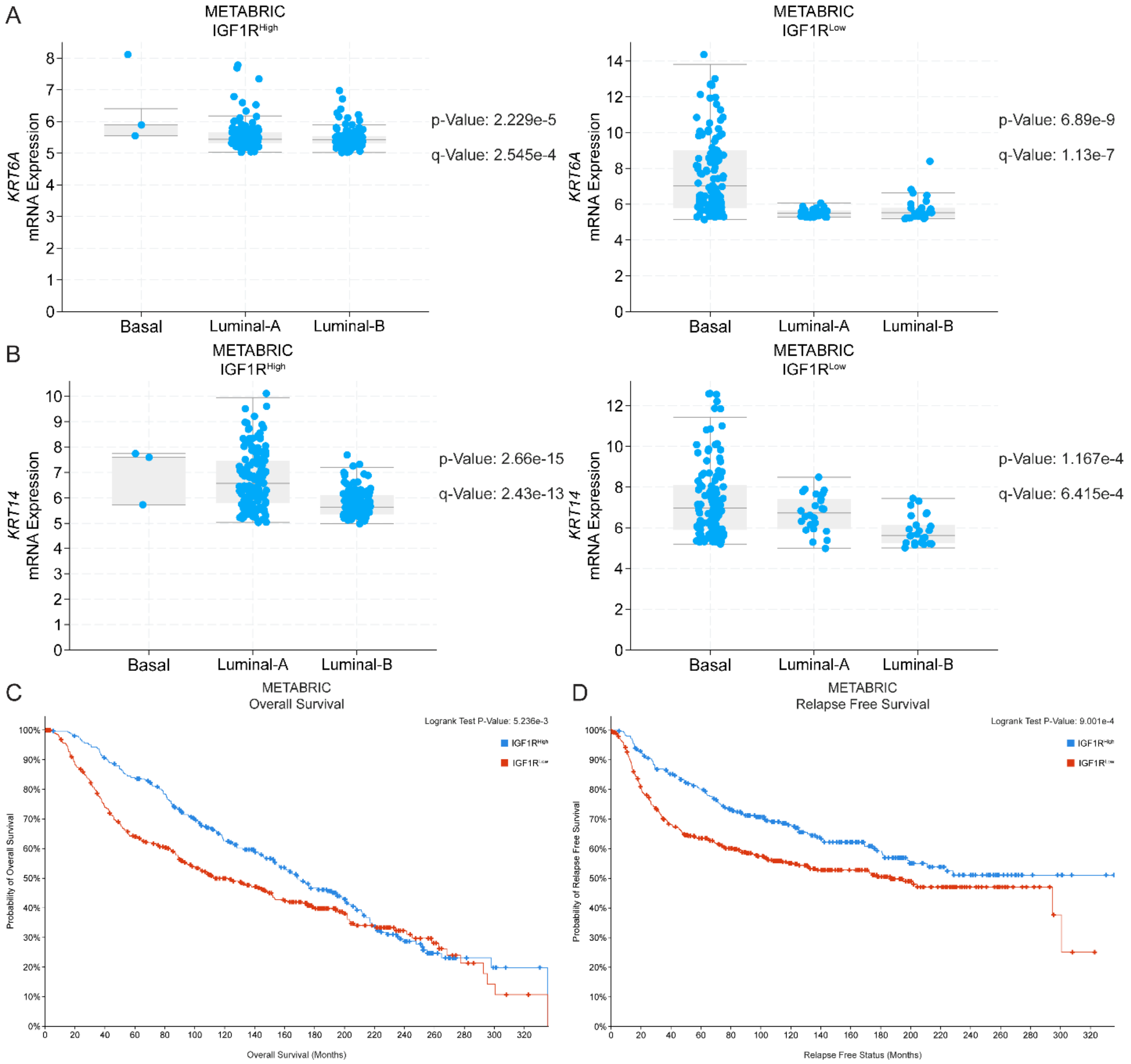

Supplemental Figure S4. cBioPortal analysis of clinical characteristics associated with low expression of IGF1R in the METABRIC human breast cancer dataset. (**A**,**B**), mRNA sequencing analysis for *KRT6A* (A) and *KRT14* (B) in IGF1R^{High} (left) vs IGF1R^{Low} (right) patients stratified by PAM50 subtype. Basal n = 3, LumA n = 149, LumB n = 124 for IGF1R^{High} and Basal n = 113, LumA n = 24, LumB = 25 IGF1R^{Low} cohorts respectively. P-values represent one-way ANOVA and q-values represent Benjamini-Hotchberg corrections with significance denoted as p < 0.05. (**C**,**D**), Kaplan-Meier curve indicating the overall (C) and relapse-free (D) survival of IGF1R^{High} (blue) vs IGF1R^{Low} (red) cohorts. IGF1R^{High}, n = 300 and IGF1R^{Low}, n = 415. Statistical significance was determined by Log-rank test and p-values < 0.05 were considered significant.
